# How synaptic function controls critical transitions in spiking neuron networks: insight from a Kuramoto model reduction

**DOI:** 10.3389/fnetp.2024.1423023

**Published:** 2024-08-09

**Authors:** Lev A. Smirnov, Vyacheslav O. Munyayev, Maxim I. Bolotov, Grigory V. Osipov, Igor Belykh

**Affiliations:** ^1^ Department of Control Theory, Lobachevsky State University of Nizhny Novgorod, Nizhny Novgorod, Russia; ^2^ Department of Mathematics and Statistics and Neuroscience Institute, Georgia State University, Atlanta, GA, United States

**Keywords:** network physiology, integrate-and-fire models, theta neurons, Kuramoto model, synaptic activation, time delay, synchronization, partial synchronization

## Abstract

The dynamics of synaptic interactions within spiking neuron networks play a fundamental role in shaping emergent collective behavior. This paper studies a finite-size network of quadratic integrate-and-fire neurons interconnected via a general synaptic function that accounts for synaptic dynamics and time delays. Through asymptotic analysis, we transform this integrate-and-fire network into the Kuramoto-Sakaguchi model, whose parameters are explicitly expressed via synaptic function characteristics. This reduction yields analytical conditions on synaptic activation rates and time delays determining whether the synaptic coupling is attractive or repulsive. Our analysis reveals alternating stability regions for synchronous and partially synchronous firing, dependent on slow synaptic activation and time delay. We also demonstrate that the reduced microscopic model predicts the emergence of synchronization, weakly stable cyclops states, and non-stationary regimes remarkably well in the original integrate-and-fire network and its theta neuron counterpart. Our reduction approach promises to open the door to rigorous analysis of rhythmogenesis in networks with synaptic adaptation and plasticity.

## 1 Introduction

Cooperative rhythms play a pivotal role in brain functioning. Fully or partially synchronized oscillations, observed across various frequency bands, underlie fundamental processes such as perception, cognition, and motor control Churchland and Sejnowski, 1992; Mizuseki and Buzsaki, 2014; Kopell et al., 2000. Extensive research has focused on the emergence of cooperative rhythms in networks of spiking and bursting neurons, encompassing synchronization Kopell et al., 2000; Brunel, 2000; Börgers and Kopell, 2003; Somers and Kopell, 1993; Izhikevich, 2007; Belykh et al., 2005; Ermentrout and Terman, 2010, partial and cluster synchronization Achuthan and Canavier 2009; Shilnikov et al., 2008; Belykh and Hasler, 2011; Schöll, 2016; Berner et al., 2021a, neural bumps Laing and Chow, 2001; Gutkin et al., 2001, and chimera states Olmi et al., 2011; Omelchenko et al., 2013.

Networks of spiking neurons with fast synaptic connections are often modeled via pulsatile on-off coupling, which sharply activates upon the arrival of a spike from a pre-synaptic cell. Such interactions are conveniently represented by networks of quadratic integrate-and-fire (QIF) models particularly suitable for large-scale simulations and analysis of cooperative dynamics Gerstner and Kistler (2002). The macroscopic dynamics of QIF networks have received extensive attention through the reduction to low-dimensional model descriptions, especially in the thermodynamic limit of infinite-dimensional networks Montbrió et al., 2015; Pazó and Montbrió, 2016; Devalle et al., 2017; Esnaola-Acebes et al., 2017; Devalle et al., 2018; Schmidt et al., 2018; Pietras et al., 2019; Montbrió and Pazó 2020; Lin et al., 2020; Gast et al., 2020; Taher et al., 2020; Byrne et al., 2022; Clusella et al., 2022; Clusella and Montbrió 2024; Ratas and Pyragas, 2018; Pyragas and Pyragas 2022, 2023; Coombes 2023; Ferrara et al., 2023. Notably, Montbrió et al. (2015) derived exact macroscopic equations for QIF networks, uncovering an effective coupling between firing rate and mean membrane potential governing network dynamics. Pietras et al. (2023) offered an analytical description of QIF network macroscopic dynamics, extending beyond the Ott-Antosen ansatz Ott and Antonsen (2008) and exploring various fast synaptic pulse profile choices. The impact of synaptic time delay on the collective dynamics of integrate-and-fire networks with sharply activated synaptic coupling, modeled by the Dirac delta function, was also extensively explored (Ernst et al., 1995; Devalle et al., 2018; Ratas and Pyragas, 2018; Pyragas and Pyragas 2022, 2023). In particular, Devalle et al. (2018) reduced a QIF model with synaptic delay to a set of firing rate equations to analyze the existence and stability of partially synchronous states. Ratas and Pyragas (2018) employed a Lorenzian ansatz to characterize macroscopic oscillations of a QIF network with heterogeneous time-delayed delta function synapses. However, there is a lack of analytical studies on the role of slower synaptic activation, potentially in the presence of time delays, in controlling critical phase transitions in QIF networks. Nevertheless, since the seminal paper by Van Vreeswijk et al. (1994), it has been recognized that slow inhibitory and excitatory synapses can reverse their roles, with slow inhibition favoring synchronization Golomb and Rinzel 1993; Terman et al., 1998; Elson et al., 2002. While predicting the exact rates of synaptic activation inducing such critical transitions in conductance-based spiking models may be challenging, analytically tractable QIF networks offer promising avenues for such exploration.

Toward this goal, this paper investigates a finite-size network of QIF neurons globally connected via a general kernel function that governs both synaptic activation and synaptic time delay. We analytically illustrate how the shape of the kernel function impacts neuron interaction, significantly altering the microscopic and macroscopic behavior of QIF networks representing Type I neuron populations. This is achieved by reducing QIF networks and their phase analog, theta neuron networks, to the Kuramoto-Sakaguchi (KS) model. Here, oscillator frequencies, coupling strength, and the Sakaguchi phase lag parameter are determined by the pulse profile’s first and second terms in the Fourier expansion. We conduct this reduction under the weak coupling assumption, utilizing the intermediate step of representing the QIF network as a generalized Winfree model, subsequently reduced to the KS model.

In our recent study Munyayev et al. (2023), we elucidated the qualitative connection between the dynamics of QIF networks incorporating synaptic dynamics and neuronal refractoriness, and the second-order Kuramoto model with high-order mode coupling. Here, we use multi-scale analysis to derive exact relationships between the QIF network with an arbitrary synaptic activation function and the KS model. Specifically, we establish explicit conditions on the parameters of the general kernel function that lead to critical transitions, determining whether the coupling is attractive or repulsive. Consequently, these conditions dictate the emergence of stable synchronization or nonstationary generalized splay states Berner et al. (2021b) and cyclops states Munyayev et al. (2023). Our analysis reveals alternating stability regions for network synchronization, dependent on both the (slow) synaptic activation and time delay. With some important caveats, this finding can be interpreted as an analogous stability criterion for synchronization in time-delayed phase oscillator networks Earl and Strogatz (2003).

Our approach serves as a connecting link between two alternative methodologies for describing macroscopic dynamics: QIF networks and theta neurons, and Winfree-type models Pazó and Montbrió, 2014; Gallego et al., 2017; Montbrió and Pazó, 2018; Pazó et al., 2019; Pazó and Gallego 2020; Manoranjani et al., 2021; Bick et al., 2020. Our KS model reduction of the generalized Winfree model with a general synaptic activation function can be seen as an extension of the work Montbrió and Pazó (2018), where a two-population Kuramoto model was derived from a network of Winfree oscillators featuring a feedback loop between fast excitation and slow inhibition.

The structure of this paper is outlined as follows. Section 2 presents the QIF network model, its theta neuron equivalent, and the general synaptic activation function. Section 3 details transforming the theta neuron model into the generalized Winfree model. We expand the pulse profile as a Fourier series and further simplify the model to the KS model using weak coupling-enabled averaging techniques. Section 4 focuses on a specific example of synaptic activation, presenting a class of kernel functions. We establish exact conditions determining whether the synaptic coupling is attractive, promoting synchronization, or repulsive, favoring splay and cyclops states. Section 5 offers numerical validation of the derived conditions and presents a comparison between the dynamics of the QIF network, the theta neuron model, and the reduced KS model. We demonstrate that the KS model accurately predicts firing rates and times, capturing the emergence of synchronization, weakly stable cyclops states, and non-stationary regimes. Section 6 contains concluding remarks and discussions.

## 2 The general QIF network and its theta neuron representation

Physiologically, excitable neurons are commonly categorized into two types. We focus here on Type I neurons, a group encompassing cortical excitatory pyramidal neurons. When subjected to a sufficiently large input stimulus, these neurons exhibit action potentials at an arbitrarily low rate, signaling the disappearance of a resting state through a saddle-node bifurcation. The canonical model used to describe Type I neurons is the QIF neuron model, which characterizes neurons’ dynamics near the spiking threshold Izhikevich (2007).

This study investigates a globally coupled network of N QIF neurons interacting through chemical synapses. Each neuron’s microscopic state is characterized by its individual membrane potential $v_{n}$ , governed by the following ordinary differential equation Izhikevich (2007):

$$\begin{array}{ccc} {\dot{v}}_{n} & =v_{n}^{2}+\eta_{n}+\varkappa S\left(t\right)\; & \text{if }v_{n}<v_{th},\\ v_{n} & =v_{r}\; & \text{if }v_{n}\geq v_{th}. \end{array}$$ (1)

Here, $\eta_{n}$ , n=1,2,…,N represents external constant currents applied to neurons, ϰ is a common synaptic weight controlling the total strength of synaptic inputs, and $S\left(t\right)$ is a time-varying input drive. When the membrane potential $v_{n}$ of the n th neuron reaches the threshold value $v_{th}$ , the neuron generates a spike, and its voltage resets to $v_{r}$ . In the absence of the input drive $\left(S\left(t\right)=0\right)$ , the intrinsic applied current $\eta_{n}=0$ places the corresponding n th neuron at a saddle-node bifurcation, marking the onset of periodic firing. Thus, if $\eta_{n}<0$ , the neuron is in the excitable regime, while if $\eta_{n}>0$ , it is in the oscillatory regime.

The last term on the right-hand side of (Eq. 1) represents synaptic interactions characterized by the coupling strength ϰ of the global synaptic drive. In general, the mean population synaptic activity $S\left(t\right)$ can be expressed by the following recurrent input equation:

$$S\left(t\right)=\frac{1}{N}\overset{N}{\underset{n^{\prime }=1}{\sum }}\;\underset{m\backslash t_{n^{\prime }}^{m}<t}{\sum }\;\overset{t}{\underset{-\infty }{\int }}\;\;d\tilde{t}\;G\left(\left(t-\tilde{t}\;\right)/\tau \right)\delta \left(\tilde{t}-t_{n^{\prime }}^{m}\right).$$ (2)

This equation accounts for relaxation processes and describes a specific type of neuron activation and its sensitivity to stimuli from other cells, including signal duration and post-spike latency. Here, $t_{n^{\prime }}^{m}$ denotes the time of the m th spike of the $n^{\prime }$ th neuron, $\delta \left(t\right)$ represents the Dirac delta function, and $G\left(t/\tau \right)$ is the normalized synaptic activation caused by a single presynaptic spike with a time scale τ . Notably, the integral transformation with the kernel $G\left(t/\tau \right)$ acts as a low-pass filter.

The QIF-neuron model (Eq. 1) describes the membrane potential $v_{n}$ and operates as a hybrid dynamical system, incorporating instantaneous resets to a base value $v_{r}$ upon spike emission. While this formulation provides a direct physical interpretation, discontinuities can pose challenges for certain applications. Fortunately, a smooth change of coordinates exists, transforming the QIF-neuron dynamics into a space where the membrane potential $v_{n}$ is represented by a phase variable $\theta_{n}$ on the unit circle. This representation captures nonlinear spike-generating mechanisms of Type I neurons, ensuring smooth solutions within a compact domain. In the limit $v_{th}=-v_{r}\to \infty$ , the transformation $v_{n}\left(t\right)=\tan\left(\theta_{n}\left(t\right)/2\right)$ Ermentrout and Kopell (1986) converts the membrane potential description of the QIF-neuron model (Eq. 1) into a canonical theta neuron model of a population of Type I neurons coupled by an excitatory or inhibitory synaptic drive. In this case, each neuron’s dynamics is governed by the following equation:

$$\frac{d\theta_{n}}{dt}=\left(1-\cos\theta_{n}\right)+\eta_{n}\left(1+\cos\theta_{n}\right)+\varkappa \left(1+\cos\theta_{n}\right)S\left(t\right),$$ (3)

where n=1,…,N represents the index of the n th neuron, and its state is characterized by the phase angle $\theta_{n}$ . We assume that the constant excitability parameters $\eta_{n}$ , akin to fixed input currents, have slightly different values across the network elements. Moreover, we consider $\eta_{n}>0$ , placing each neuron in an oscillatory regime indicative of periodic spiking. A neuron is deemed to spike or produce an action potential when $\theta_{n}$ crosses π while increasing. Consequently, in addition to a common external input $\eta_{n}$ , each cell receives stimulation from other cells. Thus, the neurons are recurrently coupled via synaptic current $S\left(t\right)$ .

The last term on the right-hand side of (Eq. 3) accounts for chemical interactions among neurons. The coupling strength ϰ is assumed to be uniform across all neurons. We model the synaptic activity $S\left(t\right)$ acting on a neuron with the following expression:

$$S\left(t\right)=\;\;\overset{t}{\underset{-\infty }{\int }}\;\;d\tilde{t}\;G\left(\left(t-\tilde{t}\;\right)/\tau \right)\;\left[\frac{1}{N}\overset{N}{\underset{n=1}{\sum }}P_{\nu }\left(\theta_{n}\;\left(\tilde{t}\;\right)\right)\right],$$ (4)

where the function

$$P_{\nu }\left(\theta_{n}\right)={\text{p}}_{\nu }{\left(1-\cos\theta_{n}\right)}^{\nu },\;\nu \in N$$ (5)

determines the shape of the pulsatile chemical synapse. The positive integer parameter ν controls the sharpness of $P_{\nu }\left(\theta_{n}\right)$ , with higher values yielding sharper peaks. Note that as ν→∞ , the smooth profile $P_{\nu }\left(\theta_{n}\right)$ converges to $P_{\infty }\left(\theta_{n}\right)$ , effectively representing δ -pulses so that

$$P_{\nu }\left(\theta_{n}\right)\overset{\nu \to \infty }{\to }P_{\infty }\left(\theta_{n}\right)=2\;{\text{C}}_{2\pi }\;\left(\theta_{n}-\pi \right)=2\;\overset{+\infty }{\underset{k=-\infty }{\sum }}\;\;\delta \left(\theta_{n}\;-\;\pi \;-\;2\pi k\right),$$ (6)

where ${\text{C}}_{T}\left(\cdot \right)$ denotes the Dirac comb with period T . In this limit and under the assumption $v_{th}=-v_{r}\to \infty$ , the theta neuron model (Eqs 3, 4) fully aligns with the QIF-neuron model (Eqs 1, 2). It is noteworthy that these models exhibit an unconventional characteristic: in contrast to conductance-based models, inhibitory δ -pulse coupling (ϰ<0) promotes synchronization, thus being considered attractive, while excitatory δ -pulse coupling (ϰ>0) is repulsive Izhikevich (2007).

Note that the limiting δ -pulse case with $P_{\infty }\left(\theta_{n}\right)$ offers a convenient framework in which function G(t) controls synaptic activation and deactivation after the instantaneous occurrence of a presynaptic action potential. The use of $P_{\infty }\left(\theta_{n}\right)$ simplifies the analytical expressions in the KS model to be more explicit and manageable (see next section). However, the general Eqs 4, 5 for synaptic input S(t) provide a more comprehensive and accurate description that captures the nuances of synaptic transmission processes. The release of neurotransmitters from the presynaptic neuron that induces a chemically activated synaptic current is non-instantaneous. Thus, the general function $P_{\nu }\left(\theta_{n}\right)$ more adequately describes this gradual neurotransmitter release that takes place as the presynaptic neuron state approaches a spike activation threshold.

In this work, we primarily focus on the pulse shape defined by (Eq. 5), originally proposed in Ariaratnam and Strogatz (2001) and widely adopted in recent studies of pulse-coupled phase oscillators O’Keeffe and Strogatz 2016; Pazó and Montbrió, 2014; Gallego et al., 2017; Pazó et al., 2019; Bick et al., 2020 and populations of theta neurons Luke et al., 2013; So et al., 2014; Laing 2014, Laing 2015; Montbrió et al., 2015; Pazó and Montbrió, 2016; Chandra et al., 2017; Goel and Ermentrout 2002; Bick et al., 2020; Pietras et al., 2023. However, our approach is directly applicable to alternative pulse shapes satisfying common properties such as unimodality, normalization, symmetry, and localization around $\theta_{n}=\pi$ and considered in previous studies Gallego et al., 2017; Pietras et al., 2023.

In the following, we explore how the shape of the kernel function $G\left(t/\tau \right)$ , governing synaptic activation, impacts the network’s collective behavior. To tackle this analytically, we will demonstrate that the model (Eqs 3, 4) consideration can be reduced to a more analytically-tractable KS model. The process involves several key steps: firstly, leveraging the assumption $\eta_{n}>0$ , we introduce an alternative phase representation for (Eqs 3, 4). Subsequently, we will derive the KS model of phase oscillators by employing multiple time scale analyses in weak chemical synaptic coupling scenarios.

## 3 Deriving the KS model from the theta-neuron model: an asymptotic analysis

We begin by assuming that each neuron operates within an oscillatory regime in the absence of interaction, i.e., $\eta_{n}>0$ . Hence, we can use a dynamical variable transformation:

$$2\tan\left(\theta_{n}/2\right)=\Omega \tan\left(\varphi_{n}/2\right),$$ (7)

where Ω is an unknown parameter to be determined subsequently. It is notable that Ω is close to $2\sqrt{\left\langle \eta_{n}\right\rangle }$ , where $\left\langle \eta_{n}\right\rangle$ denotes the mean value of the distributed external constant currents $\eta_{n}$ . However, a more precise determination of Ω is feasible for finite-size networks, as will be shown later in this section.

The transformation (Eq. 7) transitions the model (Eqs 3, 4) to an alternative phase representation:

$$\frac{d\varphi_{n}}{dt}=\Omega +\frac{2\varkappa }{\Omega }\left(1+\cos\varphi_{n}\right)\;\left(\frac{\eta_{n}-\Omega^{2}/4}{\varkappa }+S\left(t\right)\right),$$ (8)

$$S\left(t\right)=\;\;\overset{t}{\underset{-\infty }{\int }}\;\;d\tilde{t}\;G\left(\left(t-\tilde{t}\;\right)/\tau \right)\;\left[\frac{1}{N}\overset{N}{\underset{n=1}{\sum }}Q_{\nu }\left(\varphi_{n}\;\left(\tilde{t}\;\right)\right)\right],$$ (9)

where

$$Q_{\nu }\left(\varphi_{n}\right)=P_{\nu }\left(2\arctan\left(\Omega \tan\left[\varphi_{n}/2\right]\;/2\right)\right).$$ (10)

This representation remains consistent with the original description; notably, $\varphi_{n}\in \left[-\pi ,\pi \right]$ , and the occurrence of spikes for the n th neuron is still defined by $\varphi_{n}$ crossing π . However, given that in the model (Eqs 3, 4), all cells receive constant external inputs $\eta_{n}>0$ , and all units operate within the oscillatory regime, each phase $\varphi_{n}$ uniformly rotates in the absence of network interaction. Hence, the representation (Eqs 8, 9) proves more convenient for subsequent analysis. Thus, we derive the governing equation for the introduced dynamical variables $\varphi_{n}$ that may be viewed as the generalized Winfree model, which, in turn, can be reduced to the KS model. Further elaboration on this approach’s derivation and technical intricacies are presented below.

For further analysis, it is convenient to expand the symmetric pulse $Q_{\nu }\left(\varphi_{n}\right)$ into a Fourier series with respect to $\varphi_{n}$ . This expansion takes the following form:

$$Q_{\nu }\left(\varphi_{n}\right)=\;\;\overset{+\infty }{\underset{\ell =-\infty }{\sum }}\;{Q_{\nu }}_{\ell }\;e^{i\ell \varphi_{n}},\;{Q_{\nu }}_{\ell }=\frac{1}{2\pi }\overset{+\pi }{\underset{-\pi }{\int }}\;d\varphi \;e^{-i\ell \varphi }\;Q_{\nu }\left(\varphi \right),\;Q_{\nu ,-\ell }=Q_{\nu \ell }\in R.$$ (11)

The coefficients ${Q_{\nu }}_{\ell }$ in this series can be expressed analytically as follows:

$$\begin{array}{cc} {Q_{\nu }}_{\ell } & =\frac{{\text{p}}_{\nu }2^{\nu }}{\pi }\overset{\ell }{\underset{m=0}{\sum }}{\left(-1\right)}^{m}C_{2\ell }^{2m}{\left(\frac{\left|\Omega \right|}{2}\right)}^{2\left(\ell -m\right)+1}\frac{\Gamma \left(\nu +m+\frac{1}{2}\right)\Gamma \left(\ell -m+\frac{1}{2}\right)}{\Gamma \left(\nu +\ell +1\right)}\\ & \;{\times \;}_{2}F_{1}\left(\ell +1,\ell -m+\frac{1}{2};\nu +\ell +1;1-\frac{\Omega^{2}}{4}\right), \end{array}$$

where $C_{2\ell }^{2m}$ represents combinations, Γ(⋅) denotes the gamma function, ${and\;}_{2}F_{1}\left(\cdot ,\cdot ;\cdot ;\cdot \right)$ is the hypergeometric function. Specifically, for the first two Fourier series coefficients ${Q_{\infty }}_{0}$ and ${Q_{\infty }}_{1}$ of the symmetric pulse $Q_{\infty }\left(\varphi_{n}\right)$ in (Eq. 10), we obtain:

$${Q_{\nu }}_{0}={\frac{\left|\Omega \right|}{2\pi }}_{2}F_{1}\left(1,\frac{1}{2};\nu +1;1-\frac{\Omega^{2}}{4}\right),$$ (12)

$${Q_{\nu }}_{1}=\frac{\left|\Omega \right|}{4\pi \left(\nu +1\right)}[{\frac{\Omega^{2}}{4}}_{2}F_{1}\left(2,\frac{3}{2};\nu +2;1-\frac{\Omega^{2}}{4}\right)-{\left(2\nu +1\right)}_{2}F_{1}\left(2,\frac{1}{2};\nu +2;1-\frac{\Omega^{2}}{4}\right)].$$ (13)

Noteworthy, in the limit ν→∞ , i.e., for $Q_{\infty }\;\left(\varphi_{n}\right)$ , all coefficients in the Fourier series (Eq. 11) converge to the same value ${Q_{\infty }}_{\ell }={\left(-1\right)}^{\ell }\left|\Omega \right|/2\pi$ .

We proceed by assuming that the synaptic coupling is weak, allowing us to express it as $2\varkappa /\Omega =\varepsilon \kappa$ , where ε≪1 is a small parameter. Similarly, we assume that the deviations of external inputs $\eta_{n}$ from the value $\Omega^{2}/4$ are small, i.e., $\eta_{n}-\Omega^{2}/4=\varepsilon \Omega \sigma_{n}/2$ .

These assumptions enable a multiple-time scale analysis. To facilitate this analysis, we introduce a separation of time scales:

$$t_{k}=\varepsilon^{k}t,\;k=0,1,\;\ldots ,\infty$$ (14)

and represent each phase variable, $\varphi_{n}\left(t\right)$ , as an asymptotic series with respect to the small parameter ε :

$$\varphi_{n}\left(t\right)=\overset{\infty }{\underset{k=0}{\sum }}\varepsilon^{k}\varphi_{n}^{\left(k\right)}\left(t_{0},t_{1},t_{2},\ldots \right).$$ (15)

Substituting the series (Eq. 15) with times (Eq. 14) and (Eq. 11) into (Eqs 8, 9), and considering the zeroth order in ε , we obtain for $\varphi_{n}^{\left(0\right)}\left(t_{0},t_{1},t_{2},\ldots \right)$ :

$$\varphi_{n}^{\left(0\right)}\left(t_{0},t_{1},t_{2},\ldots \right)=\Omega t_{0}+\phi_{n}\left(t_{1},t_{2},\ldots \right)$$ (16)

and, taking into account (Eq. 16), for $S\left(t_{0},t_{1},t_{2},\ldots \right)$ we arrive at

$$\begin{array}{cc} S\left(t_{0},t_{1},t_{2},\ldots \right)\; & =\tau \;\overset{+\infty }{\underset{0}{\int }}\;\;d\xi \;G\left(\xi \right)\left[\frac{1}{N}\;\overset{N}{\underset{n^{\prime }=1}{\sum }}Q_{\nu }\left(\Omega t_{0}\;-\;\Omega \tau \xi \;+\;\phi_{n^{\prime }}\left(t_{1}\;-\;\varepsilon \tau \xi ,t_{2}\;-\;\varepsilon^{2}\tau \xi ,\ldots \right)\;\right.\right.\\ & \;+\left.\left.\varepsilon \varphi_{n^{\prime }}^{\left(1\right)}\left(t_{0}\;-\;\tau \xi ,t_{1}\;-\;\varepsilon \tau \xi ,\ldots \right)+\cdots \;\right)\right]\approx \frac{1}{N}\overset{N}{\underset{n^{\prime }=1}{\sum }}\overset{+\infty }{\underset{\ell =-\infty }{\sum }}G_{\ell }\;{Q_{\nu }}_{\ell }\;e^{i\ell \Omega t_{0}+i\ell \phi_{n^{\prime }}\left(t_{1},t_{2},\ldots \right)}, \end{array}$$ (17)

where each corresponding complex coefficient $G_{\ell }$ is determined as follows:

$$G_{\ell }=\tau \;\overset{+\infty }{\underset{0}{\int }}\;d\xi \;G\left(\xi \right)\;e^{-i\ell \Omega \tau \xi }.$$ (18)

In (Eq. 16), the first term $\Omega t_{0}$ describes the fast, free-running rotation of period $2\pi /\Omega$ , while the slow phase drifts induced by synaptic interaction are characterized by the set of slow variables $\phi_{n}\left(t_{1},t_{2},\ldots \right)$ . Hence, $\phi_{n}\left(t_{1},t_{2},\ldots \right)$ can be considered constant over time scales comparable to the period of the corresponding fast rotation. Consequently, the standard averaging method can be applied to derive the KS model corresponding to (Eqs 3, 4). Notably, in this case, the Sakaguchi phase shift emerges naturally due to the complex nature of the coefficient $G_{\ell }$ determined in (Eq. 18).

In accordance with the averaging procedure after substituting expression (Eq. 17) into (Eq. 8), the next step of our asymptotic approach involves considering all terms that are o(ε) and, in the first order in ε , we obtain a set of equations for $\varphi_{n}^{\left(1\right)}\left(t_{0},t_{1},\ldots \right)$ .

To eliminate the secular terms that grow without bounds as $t_{0}\to \infty$ , we impose the conditions

$$\frac{\partial \phi_{n}}{\partial t_{1}}=\sigma_{n}+\kappa {Q_{\nu }}_{0}+\frac{\kappa |G_{1}|{Q_{\nu }}_{1}}{N}\overset{N}{\underset{n^{\prime }=1}{\sum }}\cos\left(\phi_{n^{\prime }}-\phi_{n}+\arg\left(G_{1}\right)\right).$$ (19)

This yields a solution for $\varphi_{n}^{\left(1\right)}\left(t_{0},t_{1},\ldots \right)$ without the secular terms. Note that (Eq. 19) measures the rate of change of $\phi_{n}\left(t_{1},t_{2},\ldots \right)$ with respect to the slow time scale $t_{1}$ . Finally, taking into account the relation $d\phi_{n}/dt\approx \varepsilon \partial \phi_{n}/\partial t_{1}$ , we find that the dynamics of the slow phases $\phi_{n}\left(t\right)$ is approximately described by the KS model:

$$\frac{d\phi_{n}}{dt}=\omega_{n}+\frac{K}{N}\overset{N}{\underset{n^{\prime }=1}{\sum }}\sin\left(\phi_{n^{\prime }}-\phi_{n}-\alpha \right),$$ (20)

where

$$\omega_{n}=\varepsilon \sigma_{n}+\varepsilon \kappa {Q_{\nu }}_{0}=2\left(\eta_{n}-\Omega^{2}/4+\varkappa {Q_{\nu }}_{0}\right)\;/\Omega ,$$ (21a)

$$K=\varepsilon \kappa |G_{1}|{Q_{\nu }}_{1}=2\varkappa |G_{1}|{Q_{\nu }}_{1}/\Omega ,$$ (21b)

$$\alpha =-\arg\left(G_{1}\right)-\pi /2.$$ (21c)

To determine the unknown parameter Ω , we set the mean value of the intrinsic frequencies $\omega_{n}$ in the KS model (Eq. 20) to zero. Hence, the value of Ω can be found by solving the nonlinear algebraic equation:

$$\left\langle \eta_{n}\right\rangle -\Omega^{2}/4+\varkappa Q_{\nu 0}\left(\Omega \right)=0.$$ (22)

This choice of the optimal value of parameter Ω yields a better quantitative match between the numerical simulation results of the theta neuron model (Eqs 3, 4) and the KS model (Eq. 20), compared to the conventional choice of $2\sqrt{\left\langle \eta_{n}\right\rangle }$ . In the limiting case ν→+∞ , where the shape of the pulsatile chemical synapse $P_{\infty }\left(\theta_{n}\right)$ is determined by Eq. 6, the first two Fourier series coefficients ${Q_{\infty }}_{0}$ and ${Q_{\infty }}_{1}$ in (Eqs 12, 13) of the symmetric pulse $Q_{\infty }\left(\varphi_{n}\right)$ in Eqs 8, 9 converge to ${Q_{\infty }}_{0}=-{Q_{\infty }}_{1}=|\Omega |/2\pi$ and, hence, Eq. 22 for Ω can be solved analytically. This yields the simpler expressions for the parameters $\omega_{n}$ , K and α of the KS model (Eq. 20) with coefficients (Eqs 21a, 21b, 21c):

$$\omega_{n}=\frac{2\pi \left(\eta_{n}-\langle \eta_{n}\rangle \right)}{\varkappa +\sqrt{4\pi^{2}\langle \eta_{n}\rangle +\varkappa^{2}}},$$ (23a)

$$K=-\varkappa \left|G_{1}\right|/\pi ,$$ (23b)

$$\alpha =-\arg\left(G_{1}\right)-\pi /2,$$ (23c)

where the complex coefficient $G_{1}$ is determined as follows:

$$G_{1}=\tau \;\overset{+\infty }{\underset{0}{\int }}\;d\xi \;G\left(\xi \right)\;e^{-i\left(\varkappa +\sqrt{4\pi^{2}\left\langle \eta_{n}\right\rangle +\varkappa^{2}}\right)\tau \xi /\pi }.$$ (24)

Note that oscillator frequencies $\omega_{n}$ , coupling strength K , and the Sakaguchi phase lag parameter α in the KS model (Eq. 20) are explicitly defined through by the pulse profile’s first and second Fourier series terms $Q_{\nu 0}$ and $Q_{\nu 1}$ . In particular, the expressions (Eq. 23) clearly indicate that for the δ -pulses, the sign of the coupling strength K in the KS model is solely determined by and is opposite to the sign of the coupling κ in the original QIF model. The following section will show that this property carries over to the general finite-width pulses defined by (Eq. 5). For a specific class of the activation function G(t/τ) , we will also demonstrate that increasing the pulse width decreases the coupling strength K and practically does not affect the Sakachichi phase lag parameter α . The direct dependence on the properties of synaptic activation enables a straightforward assessment of the role of synaptic interactions in critical phase transitions and dynamics. Specifically, the coupling strength K and Sakachichi parameter α directly reflect the impact of synaptic activation on critical phase transitions and the synchronization behavior of the neuron population. In particular, determining whether the coupling in the theta neuron model (Eqs 3, 4) is attractive or repulsive presents a challenge due to the complexity of the system, whereas the KS model (Eq. 20) makes this process straightforward. In the subsequent section, we delve into this process, focusing on a particular synaptic activation profile.

## 4 The role of synaptic profile: a combined effect of activation, deactivation, and time delays

To demonstrate the important effects arising from the specific selection of the shape of a “low-pass filter” in synaptic activation, we examine the following class of kernel functions:

$$G\left(t/\tau \right)=\frac{{\left(t/\tau \right)}^{q}\;e^{-t/\tau }}{q!\;\tau }\;H\left(t/\tau \right),$$ (25)

where H(t/τ) is the Heaviside step function and q represents an integer parameter pivotal to the network dynamics, as will become evident subsequently. The chosen kernel corresponds to the Green’s function for a non-homogeneous linear differential equation of order (q+1) . In this case, the equation for the mean synaptic activity S(t) is generated by the (q+1) th order linear differential operator $\hat{L}={\left(\tau \;d/dt+1\right)}^{q+1}$ and contains an external signal that represents an average profile of spike pulses:

$${\left(\tau \frac{d}{dt}+1\right)}^{q+1}\;S\left(t\right)=\left\{\begin{array}{c} \frac{1}{N}\overset{N}{\underset{n^{\prime }=1}{\sum }}\;\underset{m\backslash t_{n^{\prime }}^{m}<t}{\sum }\delta \left(t-t_{n^{\prime }}^{m}\right),\;\\ \frac{1}{N}\overset{N}{\underset{n^{\prime }=1}{\sum }}P_{\nu }\left(\theta_{n^{\prime }}\left(t\right)\right),\;\\ \frac{1}{N}\overset{N}{\underset{n^{\prime }=1}{\sum }}Q_{\nu }\left(\varphi_{n^{\prime }}\left(t\right)\right).\; \end{array}\right.$$ (26)

The source term on the right-hand side of (Eq. 26) is presented in three interchangeable forms corresponding to the QIF model (Eqs 1, 2), theta neuron (Eqs 3, 4), and their averaged representation through the KS model (Eq. 20). This term can be interpreted as the population firing rate, which induces a post-synaptic current in response to the arrival of spikes. In the limit τ→0 , one might assume that the interaction between neurons becomes instantaneous. However, if the characteristic time scale τ of a post-synaptic response is not negligibly small, it becomes imperative to take into account the individual adaptation dynamics of the synaptic variable S(t) , encompassing its activation, deactivation, and time delay.

To describe the adaption dynamics of S(t) , it is common to assume that the synaptic variable S(t) follows the first-order Devalle et al., 2017; Bick et al., 2020; Afifurrahman et al., 2020; Afifurrahman et al., 2021; Pietras et al., 2023 or second-order ordinary differential equation Mohanty and Politi 2006; Zillmer et al., 2007; Bolotov et al., 2016; Chen et al., 2017, corresponding to q=0 and q=1 in (Eq. 26), respectively.

In the case q=0 , the mean population synaptic activity S(t) is governed by the standard relaxation rule. Here, when the $n^{\prime }$ th neuron fires at time $t_{n^{\prime }}^{m}$ , generating the m th spike in the form of the Dirac delta function, the mean activity S(t) instantaneously changes and subsequently decays exponentially in the absence of further firings. The parameter τ acts as a synaptic time constant.

**FIGURE 1:**
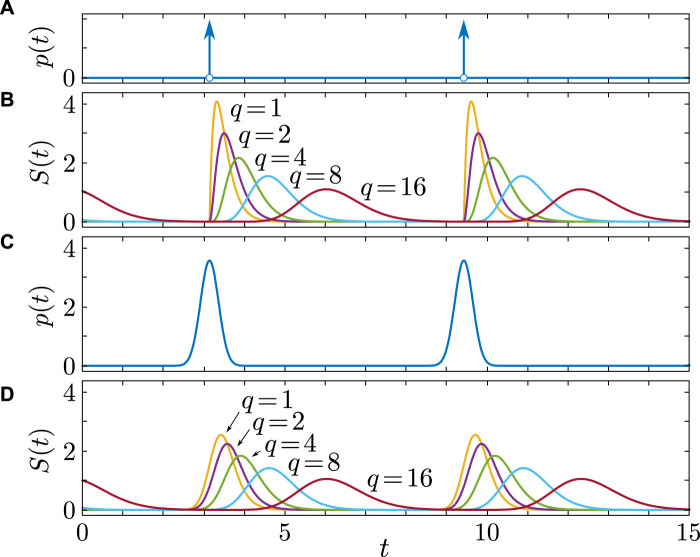
Synaptic dynamics $S\;\left(t\right)$ , defined by (Eq. 4), induced by presynaptic spikes $p\;\left(t\right)$ , taking the form of **(A,B)** $P_{\infty }\;\left(t\right)$ as defined by (Eq. 6) and **(C,D)** $P_{\nu }\;\left(t\right)$ as defined by (Eq. 5) with ν=40 and linearly increasing phase $\theta_{1}\left(t\right)=t$ (N=1) . The parameter τ=0.18 is used for all cases.

For q=1 , when the $n^{\prime }$ th neuron fires at time $t_{n^{\prime }}^{m}$ and the m th Dirac delta pulse is generated, the variable S(t) is augmented by the function $G\left(\left(t-t_{n^{\prime }}^{m}\right)/\tau \right)/N$ defined by (Eq. 25) with q=1 , coinciding with the so-called alpha-function pulse Mohanty and Politi 2006; Zillmer et al., 2007; Bolotov et al., 2016; Chen et al., 2017. For this alpha-pulse created by a spiking neuron, τ determines both the signal’s width and the time at which it attains its maximum value (Figure 1).

We extend the argument to arbitrary q and τ that make the model of synaptic adaptation (Eq. 26) encompass a broad spectrum of realistic biophysical scenarios, ranging from fast non-delayed to slow delayed activation. These scenarios include neurotransmitter release in the synaptic cleft and the opening/closing of postsynaptic ion channels, characterized by distinct time scales such as latency (time delay), rise, and decay times, as reflected in the kernel function (Eq. 25).

**FIGURE 2:**
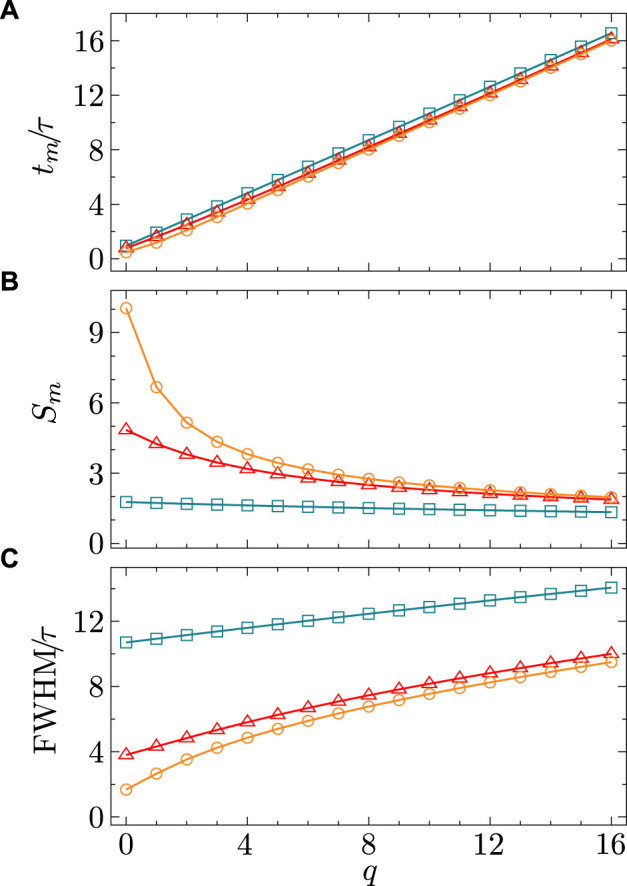
Characteristics of the synaptic dynamic profile for $S\;\left(t\right)$ (Eq. 4) as a function of q . **(A)** Peak latency, $t_{m}$ , **(B)** maximum value, $S_{m}$ , and **(C)** full width at half maximum (FWHM) , induced by spikes $P_{\nu }\;\left(t\right)$ with τ=0.1 and different ν (cyan markers – ν=10 , red markers – ν=100 , orange markers – ν=1000 ).

Our choice of the kernel function $G\left(t/\tau \right)$ for synaptic activation aligns with the gamma distribution Zwillinger (1992), a continuous probability distribution characterized by two parameters: τ>0 , the scale parameter, and q>0 , the shape parameter. This distribution reaches its maximum value at $t_{\max}=q\tau$ , with mean value $t_{\text{mean}}=\left(q+1\right)\tau$ , variance $\sigma_{t}^{2}=\left(q+1\right)\tau^{2}$ , and skewness $\gamma_{t}=2/\sqrt{q+1}$ . The skewness, reflecting the symmetry of the distribution about its mean, is maximal for the exponential case q=0 and diminishes for larger values of q , indicating increased symmetry. While both q and τ influence synaptic dynamics, q has a more significant impact on synaptic time delay than τ , whereas τ predominantly shapes the synaptic activation profile. Figures 1, 2 demonstrate how the parameters τ and q determine the time profile of the post-synaptic response and its characteristics.

Towards our objective of deriving explicit conditions for the attractiveness or repulsiveness of synaptic coupling governed by (Eq. 24) with the kernel function (Eq. 25), we calculate the complex coefficient $G_{1}$ and its modulus and argument from (Eq. 24) as follows:

$$G_{1}=\frac{1}{{\left(1+i\Omega \tau \right)}^{q+1}},\;\left|G_{1}\right|=\frac{1}{{\left(1+{\left(\Omega \tau \right)}^{2}\right)}^{\left(q+1\right)/2}},\;\arg\left(G_{1}\right)=-\left(q+1\right)\arctan\left(\Omega \tau \right),$$ (27)

where Ω is determined from (Eq. 22). While the hypergeometric function ${Q_{\nu }}_{1}$ , a factor defining the coupling strength K in the KS model (Eq. 20) with coefficients (Eq. 27), cannot be expressed via elementary functions (except for the liming δ -pulse case with ν→∞ ), it is evident that $Q_{\nu 1}\leq 0$ for ν≥0 . Consequently, the coupling strength $K=2\varkappa |G_{1}|{Q_{\nu }}_{1}/\Omega$ in the KS model (Eq. 20) for Ω>0 and the coupling strength κ in the QIF model (Eq. 1) have opposite signs. Therefore, for ϰ<0 , a positive coupling strength K corresponds to attractive coupling, provided that the Sakaguchi phase lag parameter α<π/2 . According to (Eq. 21), cos⁡α>0 if

$$\sin\;\left[\left(q+1\right)\arctan\left(\tau \Omega \right)\right]>0.$$ (28)

Solving the inequality (Eq. 28), we obtain the following $\left\lfloor q/2\right\rfloor +1$ intervals of parameters that correspond to the attractive coupling in the KS model (Eq. 20) and therefore in the QIF (Eqs 1, 2) and theta-neuron models (Eqs 3, 4) with κ<0 :

$$\begin{array}{ccc} & \tau \Omega \in D_{0}\cup \left(\tan\;\left(\frac{\left\lfloor q/2\right\rfloor }{q+1}\pi \right),+\infty \right)\;,\; & \text{for even }\left\lfloor q/2\right\rfloor ,\\ & \tau \Omega \in \left(-\infty ,-\tan\;\left(\frac{\left\lfloor q/2\right\rfloor }{q+1}\pi \right)\right)\cup D_{0},\; & \text{for odd }\left\lfloor q/2\right\rfloor , \end{array}$$ (29)

where $D_{0}=[\overset{\left\lfloor \left(q-2\right)/4\right\rfloor }{\underset{n=-\left\lfloor q/4\right\rfloor }{⋃}}\left(\tan\;\left(\frac{2n}{q+1}\pi \right),\tan\;\left(\frac{2n+1}{q+1}\pi \right)\right)]$ .

**FIGURE 3:**
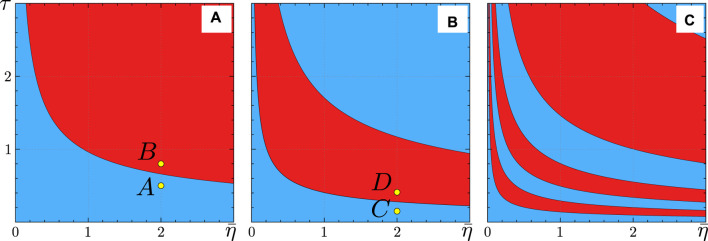
Regions of attractive (blue) and repulsive (red) coupling in the theta neuron model (Eqs 3, 4), corresponding to the KS model regions defined by (Eq. 29). The colors represent the coupling strength K sign as a function of the synaptic time constant τ and the common external input θ for a fixed q . **(A)** q=2 , **(B)** q=4 , and **(C)** q=12 . Other parameters: ϰ=−0.2π , ν=20 , $\eta_{1}=\eta_{2}=\cdots =\eta_{N}=\eta$ . The yellow points A , B , C , and D indicate the parameter values used for numerical simulations of Figures 6–9.

Figure 3 displays the regions, as defined by (Eq. 29), where the coupling in the KS model is attractive (blue) or repulsive (red). These regions exhibit an alternating pattern as functions of the synaptic time constant τ and the common external input θ for a fixed q . Increasing the parameter q , which primarily controls the time delay, makes the synaptic coupling more sensitive and results in more alternating zones. This alternating pattern of attractive and repulsive coupling resembles the stability criterion for synchronization in time-delayed phase oscillator networks, where the time delay controls the sign of the derivative of the periodic coupling function Earl and Strogatz (2003).

**FIGURE 4:**
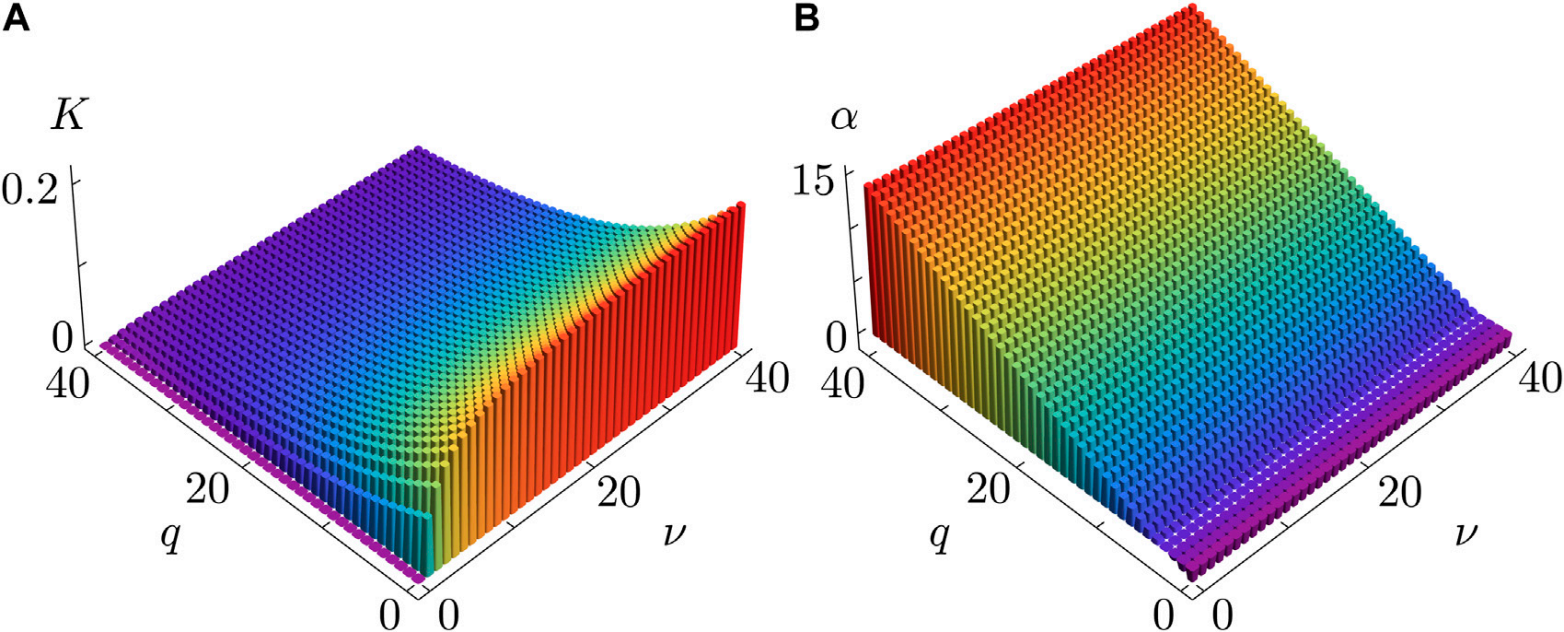
Coupling strength K **(A)** and Sakaguchi parameter α **(B)** as functions of synaptic adaptation/delay (parameter q ) and the finite pulse width (parameter ν ). The dependencies are calculated analytically via (Eq. 21) and (Eq. 27) for τ=0.15 , ϰ=−0.2π , $\bar{\eta }=2$ . The coupling strength K increases as the pulse width decreases (via increasing ν ) and decreases as synaptic adaptation slows down and experiences larger time delays (via increasing q ). The Sakaguchi parameter α is highly sensitive to q and only weakly dependent on ν .

Figure 4 provides further insight into how the synchronization properties of the KS model, critically controlled by the coupling strength K and Sakaguchi parameter α , depend on the original parameters of the QIF model. Notably, both K and α exhibit a weak dependence on ν , suggesting that the pulse width has minimal impact on synchronization dynamics, except for the case of biologically irrelevant wide pulses where ν approaches 0, making the coupling strength K significantly weaker. In contrast, both K and α are highly sensitive to variations in q , with the latter producing the alternating regions of attractive and repulsive coupling, as illustrated in Figure 3.

In the following, we offer additional evidence supporting the predictive power of the derived KS model. We show numerically that it effectively captures the emergence of robust dynamical regimes like synchronization and more intricate partially synchronized dynamics such as weakly stable cyclops states and non-stationary generalized splay states in both the QIF and theta neuron models.

## 5 Dynamical equivalence of the models: numerical validation

We conduct numerical computations using a widely accepted fifth-order Runge–Kutta method with a fixed time step of 0.01, providing additional validation for our analytical findings and predictions.

To characterize the dynamical regimes, we utilize both microscopic measures (pairwise phase differences and firing times) and macroscopic indicators such as the first- and second-order complex Kuramoto parameters Daido 1992; Skardal et al., 2011:

$$R_{\ell }\;\left(t\right)=\frac{1}{N}\;\overset{N}{\underset{n^{\prime }=1}{\sum }}\;e^{i\ell \varphi_{n^{\prime }}}=r_{\ell }e^{i\psi_{\ell }},$$ (30)

where $r_{\ell }$ and $\psi_{\ell }$ , l=1,2 define the magnitude and the phase of the ℓ th moment Kuramoto order parameter $R_{\ell }\left(t\right)$ , respectively. The first-order scalar parameter $r_{1}=|R_{1}|$ characterizes the degree of phase synchrony with $r_{1}=1$ corresponding to full phase synchrony. The second-order scalar parameter $r_{2}=|R_{2}|$ determines the degree of cluster synchrony, where $r_{2}=0$ corresponds to generalized splay states Berner et al. (2021b) and their particular case of cyclops states Munyayev et al. (2023). We will also use the firing rate, which measures the average rate at which neurons emit spikes, as another important macroscopic observable to characterize dynamical regimes. We will use the following formula Pietras et al. (2023) in simulations later in the section:

$$\rho \;\left(t\right)=\frac{1}{N}\overset{N}{\underset{n^{\prime }=1}{\sum }}\;\underset{m\backslash t_{n^{\prime }}^{m}<t}{\sum }\;\frac{1}{\Delta t}\overset{t}{\underset{t-\Delta t}{\int }}\;\;d\tilde{t}\;\delta \left(\tilde{t}-t_{n^{\prime }}^{m}\right).$$ (31)

To identify the time steps corresponding to neuron spike events in both the QIF-neuron model and the theta neuron model, we monitored the sign changes of $v_{n}\left(t\right)-v_{th}$ and $\left(\theta_{n}\left(t\right)\;\mathrm{mod}\;2\pi \right)-\pi$ , along with their time derivative signs. Subsequently, we determined the spike moment $t_{n}^{m}$ using linear interpolation within each time step.

**FIGURE 5:**
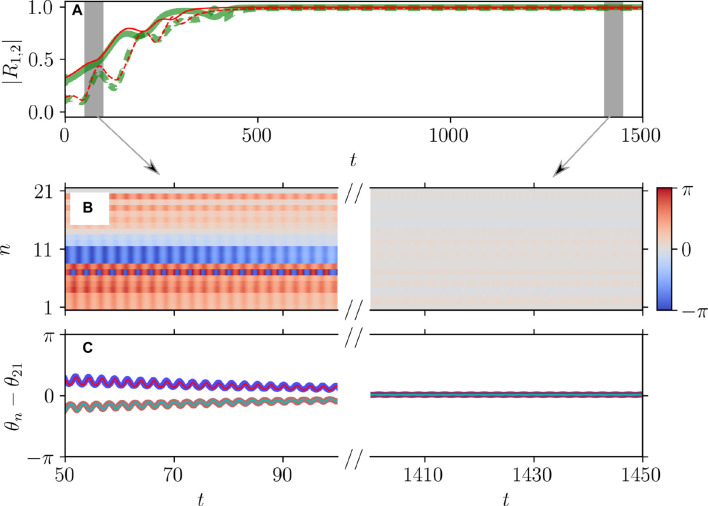
Dynamical equivalence between the theta neuron (Eqs 3, 4) and KS models (Eq. 20), demonstrated via the onset of full synchronization. **(A)** The evolution of the first $\left|R_{1}\right|$ (solid curves) and second $\left|R_{2}\right|$ (dashed curves) order parameters for the theta neuron (green curves) and KS model (red curves), including the transient period. Initial phases $\theta_{n}$ , n=1,…,N=21 are uniformly distributed over the interval [−π;π] . **(B)** The colors depict the phase differences $\theta_{n}-\theta_{21}$ in the theta neuron model converging to imperfect full synchronization, subject to mismatched parameters $\eta_{n}$ that are uniformly distributed on the segment $\left[\bar{\eta }-\delta \eta /2;\bar{\eta }+\delta \eta /2\right]$ , $\bar{\eta }=2.0$ , $\delta \eta =6\times 10^{-3}$ . **(C)** Comparison between the dynamics of the phase differences $\theta_{n}-\theta_{21}$ for $n=n_{1}=17$ (thick red curve) and $n=n_{2}=20$ (thick blue curve) in the theta neuron model and $\theta_{n}-\theta_{21}$ recalculated from phases $\varphi_{n}$ using the relation (Eq. 7) for $n=n_{1}=17$ (thin cyan curve) and $n=n_{2}=20$ (thin red curve) in the KS model. Note the perfect alignment of the phase-difference dynamics in the two models. Parameters: q=2 , ϰ=−0.2π , τ=0.5 , ν=20 , $\bar{\eta }=2.0$ correspond to point A on Figure 3.

**FIGURE 6:**
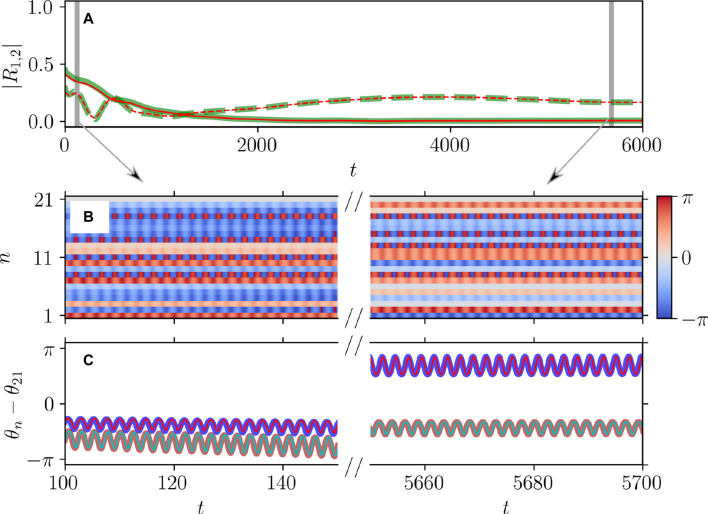
Dynamical equivalence between the theta neuron (Eqs 3, 4) and KS models (Eq. 20), demonstrated via the onset of non-stationary generalized splay state with an oscillating $|R_{1}|\approx 0$ . Notations are as in Figure 5. **(A)** The evolution of the first $\left|R_{1}\right|$ (solid curves) and second $\left|R_{2}\right|$ (dashed curves) order parameters for the theta neuron (green curves) and KS model (red curves), including the transient period. **(B)** The colors depict the phase differences θ_n_–θ_21_ in the theta neuron model. **(C)** Comparison between the dynamics of the phase differences θ_n_–θ_21_ for n = n_1_ = 17 (thick red curve) and n = n_1_ = 20 (thick blue curve) in the theta neuron model and θ_n_–θ_21_ for n = n_1_ = 17 (thin cyan curve) and n = n_1_ = 20 (thin red curve) in the KS model. Mismatch parameters $\eta_{n}$ are chosen from a uniform distribution $\left[\bar{\eta }-\delta \eta /2;\bar{\eta }+\delta \eta /2\right]$ , $\bar{\eta }=2.0$ , $\delta \eta =10^{-3}$ . Other parameters: N=21 , q=2 , ϰ=−0.2π , τ=0.8 , and ν=20 correspond to point B on Figure 3 and yield the frequency parameter Ω≈2.639 , calculated from (Eq. 22) [not shown].

**FIGURE 7:**
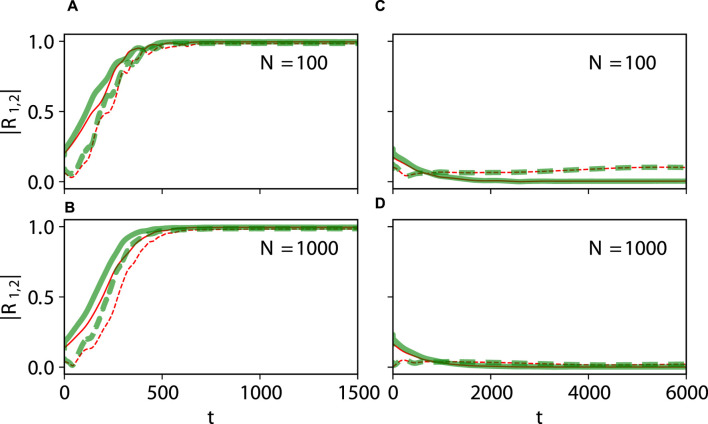
Large-size networks. Dynamical equivalence between the theta neuron (Eqs 3, 4) and KS models (Eq. 20), demonstrated via the onset of full synchronization **(A,B)** and non-stationary generalized splay state with an oscillating $|R_{1}|\approx 0$ **(C,D)** for N=100 **(A,C)** and N=1,000 **(B,D)**. Notations are as in Figures 5, 6. Parameters: q=2 , ϰ=−0.2π , τ=0.5 , ν=20 , $\bar{\eta }=2.0$ **(A,B)**; q=2 , ϰ=−0.2π , τ=0.8 , and ν=20 **(C,D)**.

Figures 5, 6 illustrate the perfect correspondence between the emergence of full synchronization and non-stationary generalized splay states in the theta neuron model (Eqs 3, 4) and the KS model (Eq. 20) within the range of attractive coupling (point A in Figure 3) and repulsive coupling (point B in Figure 3), respectively. In the case of full synchronization (Figure 5), the first-order and second-order scalar parameters (Eq. 30), $\left|R_{1}\right|$ and $\left|R_{2}\right|$ , converge to unity but cannot reach 1 due to intrinsic parameter mismatch in $\eta_{n}$ . Likewise, the first-order scalar parameter, $\left|R_{1}\right|$ , associated with the non-stationary generalized splay state oscillates closely around 0 (Figure 6). Figure 7 demonstrates that the KS model maintains its excellent predictive power for the emergence of full synchronization and non-stationary generalized splay states in large networks of 100 and 1,000 theta neurons.

**FIGURE 8:**
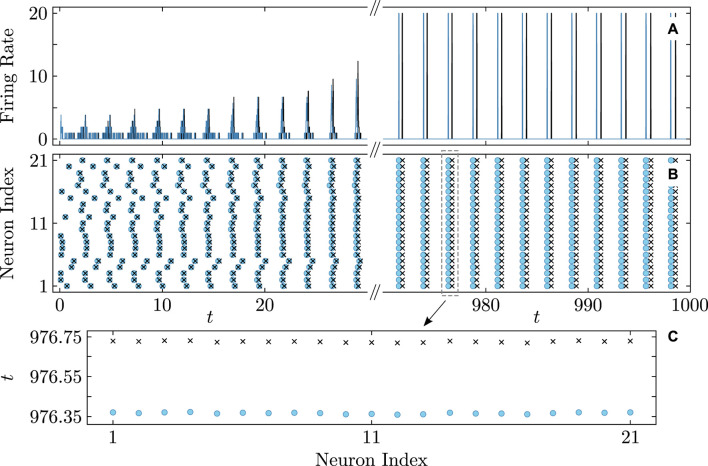
Onset of full synchronization in the QIF (Eq. 1) and KS models (Eq. 20). **(A)** Firing rate and **(B)** firing times of QIF neurons (cyan curves and round markers) and oscillators of the KS model (black curves and cross markers) with the firing times recorded at $\theta_{n}\left(t_{f}\right)=\pi$ . Each row in **(B)** represents the firing times of a neuron/oscillator. Inset **(C)** zooms-in on the firing time pattern from **(B)**. Initial conditions are as in Figure 5. Parameters: N=21 , q=4 , τ=0.15 , ϰ=−0.2π , $v_{th}=-v_{r}=10^{5}$ , $\bar{\eta }=2$ , $\delta \eta =6\times 10^{-3}$ , $\nu =10^{5}$ correspond to point C in Figure 3. The firing rate was calculated within a sliding time window of $\delta t=5\times 10^{-2}$ .

**FIGURE 9:**
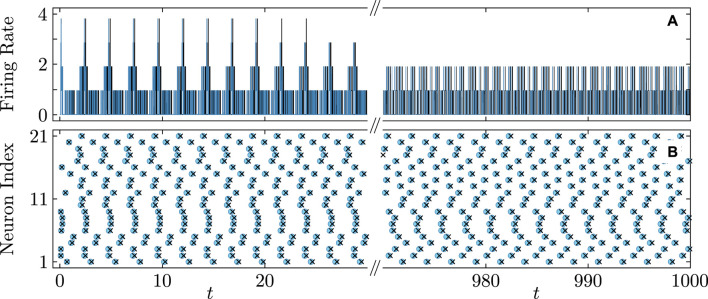
Diagram similar to Figure 8 showing a nearly perfect match for asynchronous firing rate **(A)** and firing times **(B)** in the QIF network (cyan curves and round markers) and the KS model (black curves and cross markers). Parameters: N=21 , q=4 , τ=0.41 , ϰ=−0.2π , $v_{th}=-v_{r}=10^{5}$ , $\bar{\eta }=2$ , $\delta \eta =6\times 10^{-3}$ , $\nu =10^{5}$ correspond to point D in Figure 3. Other notations and settings are as in Figure 8.

**FIGURE 10:**
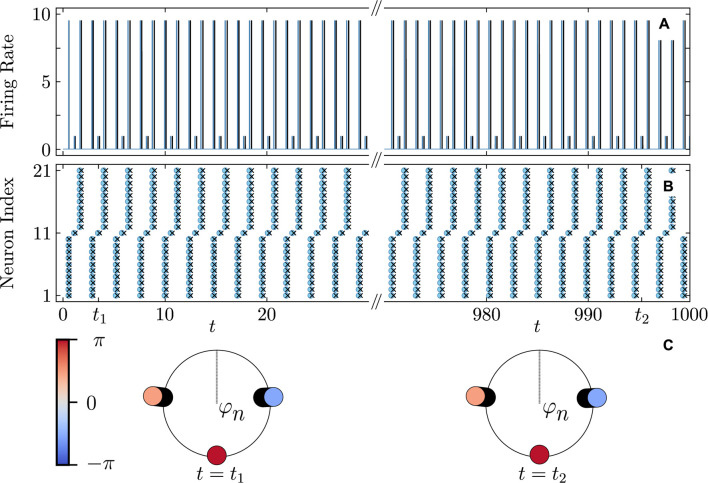
Firing rate **(A)** and firing times **(B)** of a three-cluster cyclops state in the QIF network (cyan curves and round markers) and the KS model (black curves and cross markers). **(C)** Snapshots of the cyclops state phase distributions $\varphi_{n}$ in the KS model at two time instants. The oscillators’ coloring represents their phase. The cyclops states is formed by a solitary oscillator (red) and two coherent clusters, each composed of 10 oscillators (orange and blue). The initial phases are chosen near a cyclops state. Parameters: N=21 , q=2 , τ=0.8 , ϰ=−0.2π , $v_{th}=-v_{r}=10^{5}$ , $\bar{\eta }=2$ , δη=0 , $\nu =10^{5}$ . Other notations and settings are as in Figure 8.

Figures 8–10 illustrate the remarkable agreement between cooperative dynamics in the QIF and KS models. Specifically, Figure 8 depicts the onset of full synchronization, as evidenced by synchronized firing rates and times determined via (Eq. 31). The slight discrepancy in the firing times between the QIF and KS models may stem from various sources, such as accumulated numerical errors and the approximate calculation of the frequency parameter Ω derived from (Eq. 22) for selecting the KS model parameters. Figure 9 provides evidence for the capability of the KS model to perfectly predict even non-stationary, asynchronous firing in the QIF model. Figure 10 illustrates the predictive power of the derived KS model in discerning stable complex cluster patterns like cyclops states in the QIF model. Introduced in Munyayev et al. (2023), cyclops states are formed by two distinct, coherent clusters, and a solitary oscillator reminiscent of the Cyclops’ eye. While detecting stable cyclops states can be challenging in the QIF model, the KS model provides a more convenient and constructive approach.

**FIGURE 11:**
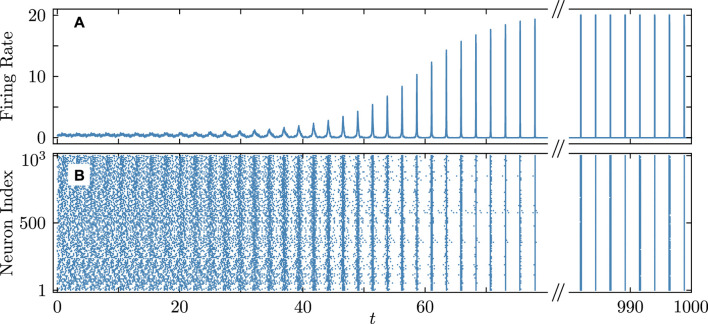
Firing rate **(A)** and firing times **(B)** in the QIF network demonstrating the transition to full synchronization starting from random initial conditions. Parameters N=1,000 , q=4 , τ=0.15 , ϰ=−0.2π , $v_{th}=-v_{r}=100$ , $\bar{\eta }=2$ , and $\delta \eta =6\times 10^{-3}$ correspond to point C in Figure 3. The simulations use the algorithm from Pazó and Montbrió (2016), which accounts for the neurons’ refractory time. The mean synaptic activation was calculated within a sliding time window of $\delta t=5\times 10^{-2}$ .

**FIGURE 12:**
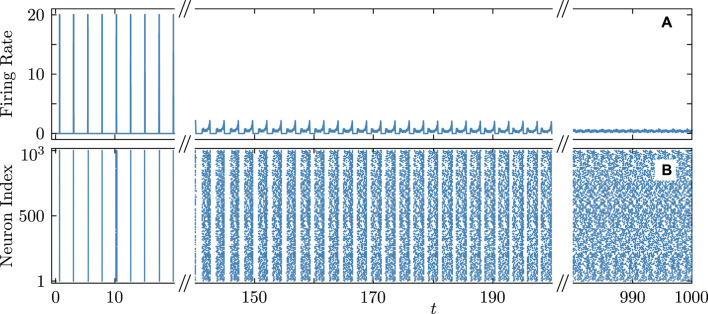
Firing rate **(A)** and firing times **(B)** in the QIF network accompanying the transition from synchronous to asynchronous dynamics. Parameters N=1,000 , q=4 , τ=0.41 , ϰ=−0.2π , $v_{th}=-v_{r}=100$ , $\bar{\eta }=2$ , and $\delta \eta =6\times 10^{-3}$ correspond to point D in Figure 3. Other notations and settings are as in Figure 11.

In the numerical calculations of relatively small-size networks of QIF neurons presented in Figures 8–10, we employed the fourth–order Runge–Kutta method using the procedure for identifying the spike events described above. To ensure better consistency between the QIF-neuron model and the theta neuron model, we used sufficiently large values of $v_{th}$ and $v_{r}$ , specifically $v_{th}=-v_{r}=10^{5}$ . We did not account for the time interval required for the membrane potential to reach ±∞ . However, this approach becomes computationally expensive for simulating large networks of QIF neurons. Therefore, in the numerical calculations presented in Figures 11, 12, we employed the algorithm described in Pazó and Montbrió (2016). This algorithm, based on the Euler method, accounts for the time it takes for the dynamic variable associated with the membrane potential to pass through the singularity ±∞ after exceeding $v_{th}$ and reach the final value $v_{r}$ . A detailed description of the algorithm and its parameters is available in the Supplemental Material for Pazó and Montbrió (2016). Figures 11, 12 confirm that the main analytical predictions for the dynamics of the QIF model remain valid for large network sizes and regardless of the calculation scheme used. Specifically, Figure 11 validates the prediction of coupling attractiveness at point C in Figure 3 and demonstrates the onset of full synchronization in the QIF network of 1,000 neurons. Similarly, Figure 12 confirms the coupling repulsiveness at point D in Figure 3 and illustrates the emergence of asynchronous dynamics. Notably, the asynchronous firing rate in the 21-node QIF network shown in Figure 9, corresponding to point D , may resemble weak coherence patterns. In contrast, its 1,000-node counterpart in Figure 12, with parameters also corresponding to point D , exhibits nearly perfect asynchronous dynamics. Our numerous additional simulations of 1,000-node QIF networks for a large set of parameters, including those near the boundary of the alternating regions of attractive and repulsive coupling in Figure 3, further validated the consistency of the predictions for cooperative dynamics and the critical transitions in the QIF networks [not shown].

## 6 Conclusions

Understanding the influence of synaptic dynamics, including activation rates, deactivation processes, and latency, on collective dynamics in neuronal networks is of significant importance. Considerable advancements have been made in analyzing the role of fast or time-delayed synapses in integrate-and-fire neuron networks. However, there remains a scarcity of analytical studies exploring the influence of slower synaptic dynamics, potentially in the presence of time delays, on controlling critical phase transitions in neuronal networks.

In this paper, we have made substantial contributions to advancing analytical methods in this field. We studied a finite-size network of QIF neurons globally interconnected via a generalized kernel function governing both synaptic activation and time delay. Our analytical exploration demonstrated how the shape of the kernel function profoundly affects neuron interaction, thereby significantly modifying the microscopic and macroscopic behavior of QIF networks. To achieve this, we reduced the QIF and theta neuron network models to the Kuramoto–Sakaguchi model. In this model, oscillator frequencies, coupling strength, and the Sakaguchi phase lag parameter are determined by the Fourier terms of the pulse profile series expansion.

We established exact conditions determining whether synaptic coupling is attractive, fostering synchronization, or repulsive, promoting splay and cyclops states. Furthermore, we demonstrated a remarkable correspondence between the dynamics of the derived KS model and the original QIF and theta neuron models. Specifically, the KS model accurately predicted firing rates and times, capturing the emergence of synchronization, weakly stable cyclops states, and non-stationary regimes in the QIF model. Our reduction approach complements the work by Ratas and Pyragas (2018), which assumed a Lorentzian distribution of the input currents and employed the thermodynamic limit to reduce the QIF model with time-delayed coupling to macroscopic equations that characterize the mean membrane potential, the spiking rate, and the mean synaptic current. The bifurcation analysis of these macroscopic equations, performed in Ratas and Pyragas (2018), revealed alternating parameter regions where the QIF network exhibits macroscopic self-oscillations as a function of input current heterogeneity and time-delayed coupling. While sharing some similarities and goals, our approach is fundamentally different. It reduces finite-size QIF networks with an arbitrary current distribution and arbitrary synaptic activation function G(t/τ) to the finite-size microscopic KS model. This allows for characterizing fine dynamical patterns, including cluster and cyclops states, which might be out of reach for a macroscopic description. In light of this, our reduction approach to an analytically tractable Kuramoto model holds promise in facilitating constructive analysis of rhythmogenesis in QIF networks. By utilizing the reduced KS model, a variety of methods and analytical machinery can be applied to the analysis of collective dynamics in QIF models, including the constructive selection of complex patterns, such as chimeras, whose existence and emergence might be easier to deduce from the reduced Kuramoto model description. Furthermore, the reduction approach holds the potential for extensions to incorporate synaptic adaptation, Hebbian learning, and a complex network structure by employing node-degree block approximation.

## Data Availability

The original contributions presented in the study are included in the article/supplementary material, further inquiries can be directed to the corresponding author.
